# Pinpointing retrovirus entry sites in cells expressing alternatively spliced receptor isoforms by single virus imaging

**DOI:** 10.1186/1742-4690-11-47

**Published:** 2014-06-16

**Authors:** Sergi Padilla-Parra, Mariana Marin, Naoyuki Kondo, Gregory B Melikyan

**Affiliations:** 1 Division of Structural Biology, University of Oxford, The Wellcome Trust Centre for Human Genetics, Headington, Oxford OX3 7BN, UK; 2 Division of Pediatric Infectious Diseases, Emory University Children’s Center, Atlanta, GA 30322, USA; 3 Children’s Healthcare of Atlanta, Atlanta, GA 30322, USA; 4 Current address: Department of Molecular Genetics, Institute of Biomedical Science, Kansai Medical University, Osaka, Japan

**Keywords:** Avian sarcoma and leukosis virus, Single particle tracking, Acid-induced virus fusion, Fusion kinetics, Fusion pore, Endosome maturation, Rab proteins

## Abstract

**Background:**

The majority of viruses enter host cells *via* endocytosis. Current knowledge of viral entry pathways is largely based upon infectivity measurements following genetic and/or pharmacological interventions that disrupt vesicular trafficking and maturation. Imaging of single virus entry in living cells provides a powerful means to delineate viral trafficking pathways and entry sites under physiological conditions.

**Results:**

Here, we visualized single avian retrovirus co-trafficking with markers for early (Rab5) and late (Rab7) endosomes, acidification of endosomal lumen and the resulting viral fusion measured by the viral content release into the cytoplasm. Virus-carrying vesicles either merged with the existing Rab5-positive early endosomes or slowly accumulated Rab5. The Rab5 recruitment to virus-carrying endosomes correlated with acidification of their lumen. Viral fusion occurred either in early (Rab5-positive) or intermediate (Rab5- and Rab7-positive) compartments. Interestingly, different isoforms of the cognate receptor directed virus entry from distinct endosomes. In cells expressing the transmembrane receptor, viruses preferentially entered and fused with slowly maturing early endosomes prior to accumulation of Rab7. By comparison, in cells expressing the GPI-anchored receptor, viruses entered both slowly and quickly maturing endosomes and fused with early (Rab5-positive) and intermediate (Rab5- and Rab7-positive) compartments.

**Conclusions:**

Since the rate of low pH-triggered fusion was independent of the receptor isoform, we concluded that the sites of virus entry are determined by the kinetic competition between endosome maturation and viral fusion. Our findings demonstrate the ability of this retrovirus to enter cells *via* alternative endocytic pathways and establish infection by releasing its content from distinct endosomal compartments.

## Background

Many pathogens, including enveloped and non-enveloped viruses, use distinct endocytic pathways to infect their host cells [1-4]. Viral entry routes have been traditionally studied by blocking one or more endocytic pathways and/or preventing endosome maturation (reviewed in [4]). These strategies include pharmacological interventions, silencing the expression of proteins involved in vesicular trafficking or over-expression of their dominant-negative mutants. The main limitation of these approaches is that they often have off-target effects by altering key cellular functions. For instance, agents that raise endosomal pH block entry of viruses whose fusion is triggered by acidic conditions, but can also interfere with pH-independent steps of viral entry [5-7] due to the coupling between endosome maturation and acidification of their lumen [8-11]. Another widely used approach to elucidate the viral entry pathways is based on virus colocalization with endosomal cargo or markers for specific intracellular compartments in fixed cells. This approach suffers from poor spatial resolution (optical microscopy) or lack of information regarding specific proteins (conventional electron microscopy). Although immunogold labeling and super-resolution fluorescence imaging address these issues, the fact that the majority of viruses fails to establish infection and can thus be trafficked through non-productive pathways limits the utility of these approaches.

The pioneering work by A. Helenius, X. Zhuang and others introduced real-time single virus tracking in live cells to delineate their entry pathways [12-18]. When combined with imaging of formation and maturation of endosomal compartments, single particle tracking is a powerful means to define the sites of virus entry [14,15,19-21]. Furthermore, single particle tracking and detection of virus-endosome fusion provides the much needed evidence for productive entry culminating in the release of viral genome [19,22,23]. Visualization of the influenza virus co-trafficking with markers for early (Rab5) and late (Rab7) endosomes [8-10,24], along with detection of the lipid mixing step of viral fusion, revealed that this virus preferentially enters a subset of quickly maturing vesicles and fuses with maturing endosomes [19]. Imaging of Dengue virus co-trafficking with Rab5- and Rab7-positive endosomes suggests that the virus initiates lipid mixing in late endosomes [14], which have been implicated as sites of Dengue entry [14,21,25].

While the lipid mixing activity is a convenient readout for membrane fusion, strictly speaking, lipid mixing demonstrates the progression to a hemifusion stage, which is defined as the merger of contacting leaflets without the formation of a fusion pore [26,27]. There are, however, examples of hemifusion not culminating in complete viral fusion or infection [6,23,25,28-32]. Thus, lipid mixing alone, without the detection of a viral content release into the cytoplasm may not reveal the actual entry sites into cells. This consideration highlights the importance of detecting complete viral fusion as it occurs in endosomes to pinpoint the sites of virus entry.

We have developed virus labeling and time-resolved imaging techniques to track single virus movement in cells and visualize full fusion events [22,23,30,33-36]. In these experiments, fusion is detected based on the viral content release, which is a proxy for productive entry. The Avian Sarcoma and Leukosis Virus (ASLV) requires two consecutive triggers/cues to undergo fusion – priming by cognate receptors on the cell surface and low pH-dependent fusion in endosomes [33,37,38]. The robust fusion with endosomes mediated by ASLV Env and the ability to control and synchronize these events [34,35,39,40] make ASLV a valuable model system for studies of viral entry. In addition, the subgroup A virus (ASLV-A) can effectively utilize two alternatively spliced isoforms of its TVA receptor, TVA800 (GPI-anchored receptor) and TVA950 (transmembrane receptor) [41,42]. Importantly, accumulating evidence implies that these TVA isoforms direct ASLV-A entry through alternative endocytic pathways [22,34,35,39].

Simultaneous pH measurements in virus-carrying vesicles and imaging of the resulting content release from single ASLV-A pseudoviruses showed that fusion occurred at pH around 6.0 [36]. The mildly acidic pH typical for early endosomes [43] suggests that ASLV-A enters cells from these compartments. Here, we explicitly tested this conjecture by visualizing ASLV-A co-trafficking and fusion with intracellular compartments tagged with fluorescent Rab5 and Rab7 proteins, the canonical markers for early and late endosomes, respectively (e.g. [8,19,44]). Single virus imaging showed that ASLV-A fused with compartments positive for Rab5 alone or both Rab5 and Rab7, thus demonstrating that the virus can enter cells from both early or intermediate (maturing) compartments, respectively. Interestingly, ASLV-A preferentially fused with early endosomes in cells expressing TVA950, while it was equally likely to fuse with early and intermediate endosomes in cells expressing TVA800. The predominant entry from early endosomes in TVA950 cells was not due to the faster kinetics of low pH-dependent fusion in these cells compared to cells expressing TVA800. The transmembrane receptor appears to direct ASLV-A to slowly maturing endosomes where fusion tends to occur prior to accumulation of Rab7. By contrast, the nearly identical rates of fusion and endosome maturation appear to control non-selective ASLV-A fusion with early and intermediate compartments in TVA800 cells. These findings show that ASLV-A can enter cells from distinct intracellular compartments and that the sites of entry are determined by the receptor isoforms. To our knowledge, this is the first direct demonstration of complete virus fusion with specific endosomal compartments, using time-resolved single virus imaging in living cells.

## Results

### Visualization of single virus co-trafficking with endosomal markers and viral content release

We have previously visualized single virus entry and fusion with cells using particles containing a genetically encoded releasable content marker [22,23,30,33-36]. Viral fusion leads to the loss of fluorescence signal owing to the dilution of a fluorescent marker in the cytoplasm. To eliminate false-positive events due to the particle deviation from the focal plane, viruses were co-labeled with a reference marker incorporated either into the viral membrane or into the core. This enables reliable detection of fusion based upon disappearance of a viral content marker, but not of a non-releasable reference marker.

**Figure 1:**
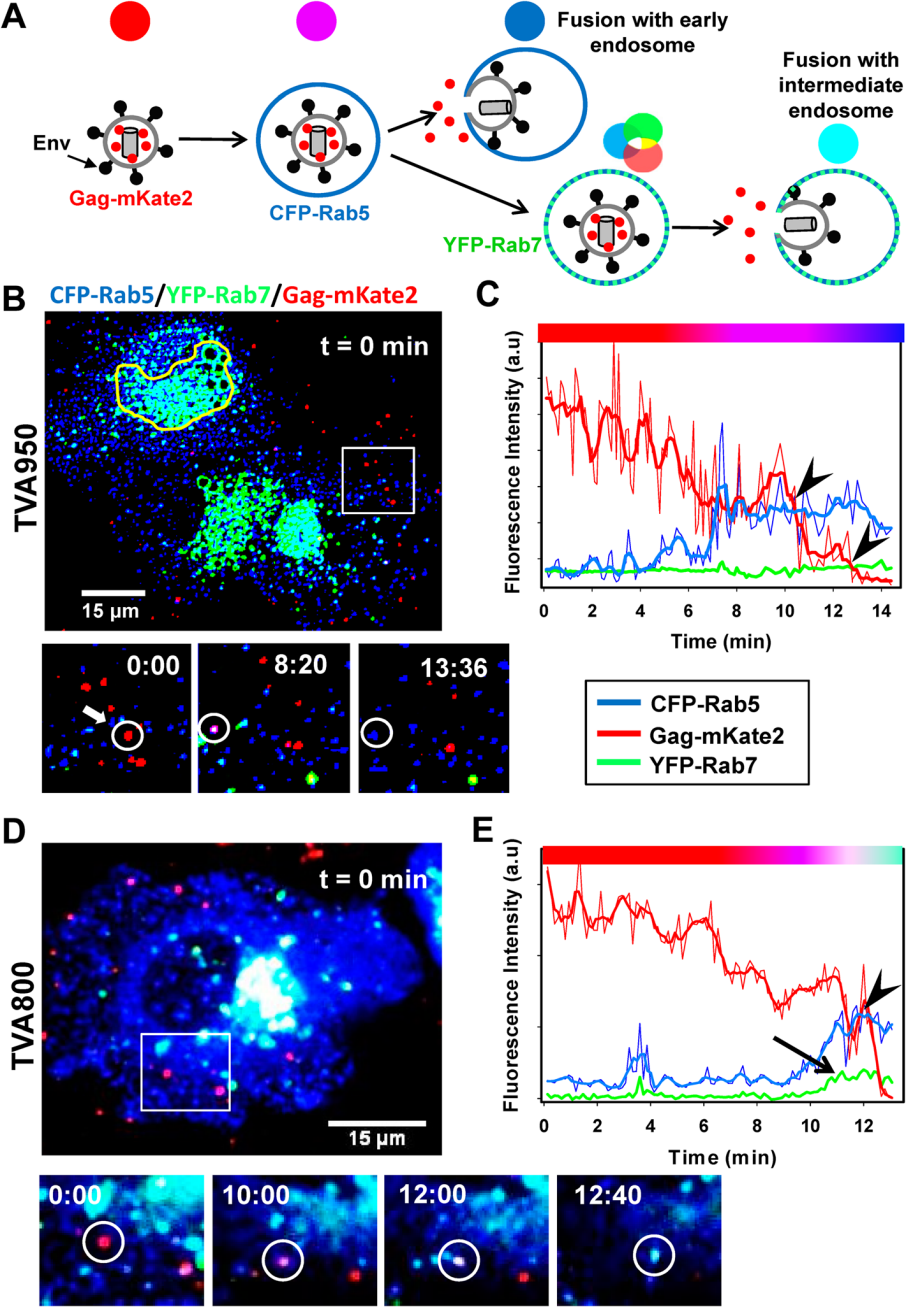
**ASLV-A trafficking and fusion with early and intermediate endosomes. (A)** Illustration of ASLV-A (Gag-mKate2-labeled, red) fusion with early endosomes (CFP-Rab5^+^, blue) and intermediate endosomes (YFP-Rab7^+^, green). Changes in pseudocolor upon virus entry and fusion with these endosomes are illustrated by small circles. **(B-E)** Fusion of Gag-mKate2-labeled ASLV-A (red) with TVA950 **(B, C)** and TVA800 **(D, E)** cells co-transfected with CFP-Rab5 (blue) and YFP-Rab7 (green). Viruses were pre-bound to cells in the cold and their internalization and fusion were initiated by shifting to 37°C. Panels **B** and **C** illustrate ASLV-A fusion with an early endosome of a TVA950 cell and panels **D** and **E** show fusion with an intermediate endosome of a TVA800 cell. The boxed areas in B and D are enlarged in the image panels below. Yellow contour in B marks highly fluorescent perinuclear area. **(C, E)** The fluorescence intensities of viral and endosomal markers as a function of time were obtained by single particle tracking. The mKate2 channel was used to track particles before they colocalized with CFP-Rab5, and the CFP channel was used for particle tracking afterwards. The gradual decrease of the mKate2 signal preceding the final drop to the background level (panels **C** and **E**) was likely caused by the particle deviation from the focal plane, but not by photobleaching (see Additional files 4 and 5: Figures S4 and S5). mKate2 release is marked by arrowheads in C and E. The viral content release in C occurred in two steps, suggesting transient closure of a nascent fusion pore [22,34]. An arrow in panel E marks the appearance of the YFP-Rab7 signal. The colored horizontal bars above the graphs show the pseudocolor changes associated with virus colocalization and fusion with early or intermediate endosomes.

In order to identify the point of virus entry into a cell, one needs to simultaneously image double-labeled viruses in cells co-expressing fluorescent markers for early (Rab5) and late (Rab7) endosomes. Such 4-color imaging experiments are technically challenging due to the spectral overlap between fluorescent proteins and poor signal/background ratio in single virus experiments. We therefore imaged the viral content release from single-labeled particles (without a reference marker) following their entry into intracellular compartments labeled with fluorescent Rab5 and Rab7 (Figure 1A). Here, fluorescent Rabs bound to virus-carrying endosomes serve as reference signals for reliable detection of the loss of viral content upon fusion. Coordinated virus movement with puncta positive for either Rab5 or Rab7 greatly diminishes the possibility of virus disappearance through departure from the focal plane or sudden lateral movements. Following the convention (e.g. [8,19]), we operationally define early endosomes as Rab5^+^/Rab7^-^, intermediate/maturing endosomes as Rab5^+^/Rab7^+^, and late endosomes as Rab5^-^/Rab7^+^ compartments.

ASLV-A appears to be internalized through different endocytic pathways in cells expressing the TVA800 or TVA950 isoforms [22,34,35,39]. We therefore hypothesized that these receptor isoforms direct virus entry from distinct compartments. To test this hypothesis, we visualized entry and fusion of content-labeled ASLV-A pseudoviruses into CV-1 cells stably expressing either TVA800 or TVA950 receptor [35] and co-transfected with CFP-Rab5 and YFP-Rab7. In order to minimize the disruptive effects of Rab overexpression which leads to the formation of aberrantly large endosomes (e.g., [19,45] and Figure 1B, yellow contour), only cells expressing low to moderate levels of both markers (Additional file 1: Figure S1) were selected for analysis. These cells exhibited punctate Rab5 and Rab7 fluorescence pattern, which enabled the visualization of virus co-trafficking with these compartments (Figure 1). Quantitative analysis showed that the Rab5 and Rab7 expression levels in TVA800 and TVA950 cells selected for imaging the virus fusion were statistically identical and significantly lower than in transfected cells excluded from analyses (Additional file 1: Figure S1).

Pseudoviruses were visualized by incorporating the Gag-mKate2 chimera, which yielded free mKate2 protein trapped within the viral envelope upon virus maturation, thus enabling the detection of virus-endosome fusion [36]. Viral particles were bound to cells by spinoculation at 4°C. Virus input was adjusted, so that equal number of pseudoviruses attached per each TVA800 and TVA950 cell (10 ± 3 and 10 ± 6 particles, respectively, P = 0.8). ASLV-A uptake and fusion were initiated by quickly raising the temperature to 37°C. Virus co-trafficking with endosomes was defined as a >80% overlap between the particle and one or both endosomal markers for 5 or more consecutive images, during which time the particle traveled at least 1 μm (for details, see Methods and Additional file 2: Figure S2).

### TVA isoforms mediate ASLV-A entry from distinct endosomal compartments

We found that the majority of cell-bound pseudoviruses co-trafficked with endosomal markers in TVA800 and in TVA950 cells (Figure 1 and Additional file 3: Figure S3). The remainder of particles did not meet the co-trafficking criterion, perhaps due to their delayed uptake and/or undetectably low amounts of fluorescent Rabs on some intracellular compartments. Entry of mKate2-labeled pseudoviruses (red) into early CFP-Rab5^+^ (blue) endosomes and then into intermediate compartments positive for both CFP-Rab5 and YFP-Rab7 (green) was manifested in pseudocolor changes from red to purple (red/blue) and, in some cases, from purple to whitish (red/blue/green, Figure 1B-E and Additional files 4 and 5: Movies S1 and S2).

At least two patterns of Rab5 acquisition were observed – merger with an existing Rab5^+^ endosome (Figure 1B,C and Additional files 6 and 7: Figures. S4A and S5A) and gradual accumulation of this marker (Figure 1D,E and Additional files 6 and 7: Figures S4B and S5B). Abrupt acquisition of Rab5 was seen for 15 out of 22 fusing particles in TVA950 cells and for 9 out of 18 fusing particles in TVA800 cells. However, this difference in the mode of endosome maturation between the two cell lines was not significant (P = 0.243). The pattern of Rab7 accretion was similar to that of Rab5: stepwise appearance due to fusion between virus-carrying vesicles with Rab5^+^/Rab7^+^ endosomes (Additional file 6: Figure S4A) or gradual accumulation (Figure 1D,E, Additional file 6: Figure S4B and Additional file 7: Figure S5A-C). Abrupt vs. gradual acquisition of Rab7 by endosomes harboring fusion-competent viruses was equally likely to occur in either cell line: 7 out of 18 (TVA800) and 12 out of 22 events (TVA950, P = 0.554).

**Figure 2:**
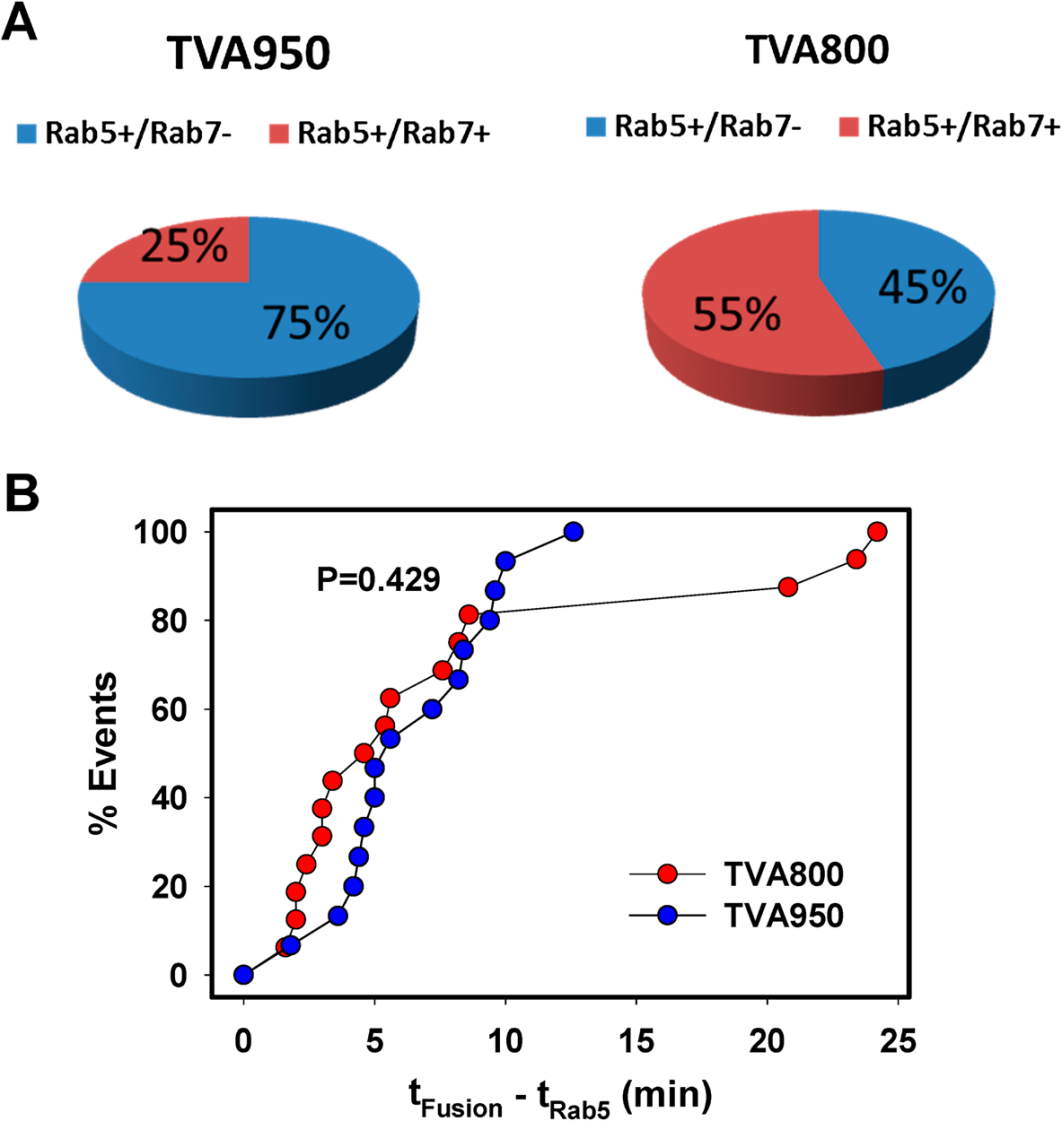
**Analysis of the extent and kinetics of ASLV-A fusion with endosomes of cells expressing alternative TVA isoforms. (A)** The distributions of ASLV-A fusion with early and intermediate endosomes in TVA950 (n = 15) and TVA800 (n = 16) cells, as determined by single particle tracking (see Figure 1 for details). **(B)** Kinetics of ASLV-A fusion with TVA800 and TVA950 cells following the virus entry into early endosomes. The time intervals from appearance of the CFP-Rab5 signal to release of the viral content (t_Fusion_ – t_Rab5_) were measured and plotted as cumulative distributions.

Almost 50% of pseudoviruses residing in Rab5^+^ or Rab5^+^/Rab7^+^ compartments underwent fusion, as evidenced by the release of viral content into the cytosol (Additional file 3: Figure S3). We observed ASLV-A fusion in both early and intermediate compartments (Figure 1). However, in TVA950 cells, content release typically occurred from early endosomes before detectable accumulation of Rab7 (Figure 1B,C and Additional file 6: Figure S4C), whereas fusion with TVA800 cells occurred in both early and intermediate compartments (Figure 1D,E and Additional file 7: Figure S5C). As shown in Figure 2A, a significantly greater fraction of pseudoviruses fused with early Rab5^+^/Rab7^-^ endosomes in TVA950 cells (n = 22) compared to TVA800 cells (n = 18, P = 0.033 based on the χ^2^ test). Notably, irrespective of the TVA isoform, all fusion events occurred in Rab5^+^ compartments – either early or intermediate endosomes.

We next asked whether the reason for the preferential ASLV-A fusion with early endosomes in TVA950 cells was due to the faster kinetics of fusion following its entry into early endosomes as compared to TVA800 cells. The kinetics of pseudovirus fusion was assessed by measuring the lag time between the Rab5 acquisition and viral fusion (t_Fusion_ - t_Rab5_, Figure 2B). This analysis revealed that the post-Rab5 acquisition kinetics of fusion with TVA800 and TVA950 cells were indistinguishable. Thus, the preferential ASLV-A entry from early endosomes of TVA950 cells could not be explained by faster fusion compared to TVA800 cells. The above results implicate slower maturation of virus-carrying endosomes in TVA950 cells as the reason for the predominant content release from early endosomes.

### ASLV-A enters from slowly maturing endosomes in TVA950-expressing cells

**Figure 3:**
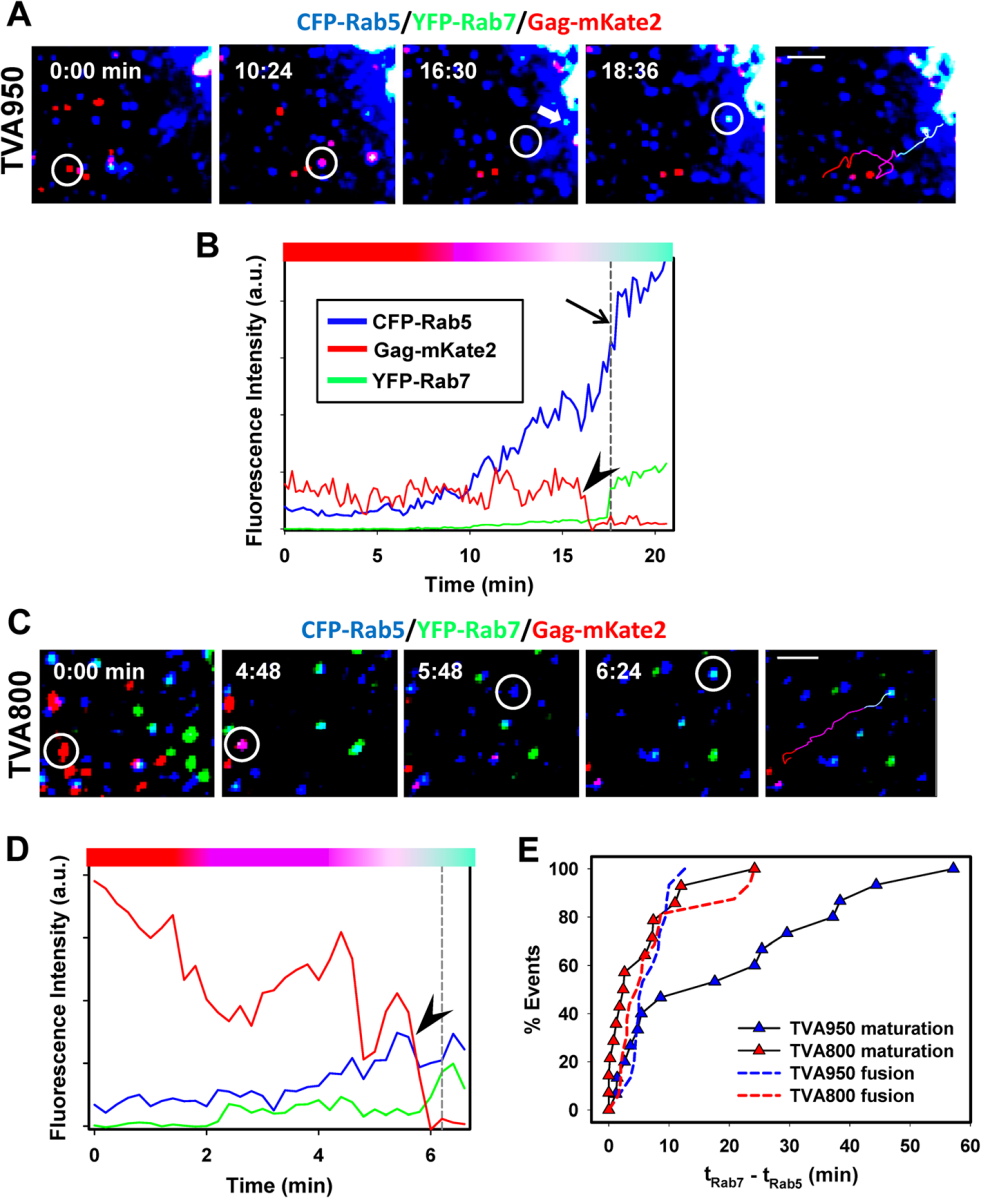
**Post-fusion maturation of ASLV-A-carrying endosomes. (A, B)** ASLV-A (red) fusion with an early (CFP-Rab5^+^) endosome followed by heterotypic fusion with an intermediate (CFP-Rab5^+^/YFP-Rab7^+^) endosome in a TVA950 cell marked by arrows in panels A and B. The last image panel shows the virus trajectory colored according to the color changes corresponding to entry into an early endosome, virus fusion and then heterotypic fusion with another endosome. Scale bar 10 μm. **(C, D)** ASLV-A (red) fusion with an early (CFP-Rab5^+^, blue) endosome of a TVA800 cell followed by acquisition of YFP-Rab7 (green). The last image panel shows the virus trajectory colored according to the color changes corresponding to entry into an early endosome, virus fusion and then heterotypic fusion with another endosome. Scale bar 5 μm. **(B, D)** The points of mKate2 release are marked by arrowheads. The vertical dashed lines mark the acquisition of the above-threshold Rab7 signal. The colored horizontal bars above the graphs reflect the pseudocolor changes associated with virus colocalization and fusion with early or intermediate endosomes. **(E)** The maturation kinetics for endosomes carrying fusion-competent ASLV-A pseudoviruses in TVA800 and TVA950 cells. The lag times between accumulation of Rab5 and Rab7 signals (t_Rab7_ – t_Rab5_) were measured, as described in Methods and plotted as cumulative distributions. To aid comparison, the kinetics of ASLV-A fusion after entry into Rab5^+^ endosomes (t_Fusion_ – t_Rab5_) are re-plotted from Figure 2B (respectively colored dashed lines).

The existence of distinct pools of endosomes, differing in their maturation rates and mobility has been reported previously [19]. In this study, maturation of early endosomes has been defined as the time interval between the appearance of Rab5 and Rab7 signals (t_Rab7_ – t_Rab5_). We also used the conversion time from early to intermediate compartments as a measure of the rate of maturation of virus-carrying endosomes. For fusion events occurring after the appearance of the Rab7 signal, particle tracking was performed using the mKate2 channel (Figure 1), whereas the time of endosomal maturation for fusion events preceding the Rab7 accumulation was obtained by tracking the Rab5 channel (Figure 3A-D and Additional files 8 and 9: Movies S3 and S4). The maturation times of virus-harboring endosomes were significantly slower in TVA950 compared to TVA800 cells (Figure 3E, P < 0.02). This finding demonstrates that ASLV-A preferentially enters from slowly maturing compartments in TVA950 cells, whereas this virus appears equally distributed between slowly and quickly maturing endosomes in TVA800 cells. Taken together, our results imply that the slower maturation kinetics of ASLV-A-containing endosomes in TVA950 cells accounts for the preferential fusion with early endosomes (Figure 2A).

### ASLV-A entry into late endosomes is delayed

Pseudoviruses that co-trafficked with endosomal markers, but did not fuse, enabled the visualization of ASLV-A transport to the perinuclear space. Almost without exception, unfused viruses resided in Rab5^+^/Rab7^+^ compartments of TVA800 and TVA950 cells at the end of imaging experiments. This was because virus-carrying intermediate endosomes did not lose Rab5 for as long as we could track them (Figure 1, Additional files 6 and 7: Figures S4 and S5, and Additional file 10: Movie S5), except for one particle in a TVA800 cell (Additional file 7: Figure S5A). Considering that the formation of late endosomes is associated with loss of Rab5 [9,10,24,46], our results indicate that virus-carrying compartments do not fully mature. The lack of full endosome maturation could be due to subversion/disruption of normal endosomal trafficking by ASLV-A. Alternatively, the brightly fluorescent perinuclear area (e.g., yellow contour in Figure 1B) could mask the loss of Rab5 following the virus entry into late endosomes.

### Virus entry into early endosome correlates with acidification of endosomal lumen

As indicated above, all viral fusion events (disregarding those that did not colocalize with any endosomal markers) occurred within Rab5^+^ spots, i.e. in early or intermediate endosomes (Figures 1, 2, 3). We thus simplified the imaging experiment by expressing only Rab5 as a reference marker, which allowed the usage of pseudoviruses co-labeled with Gag-mKate2 (the content marker) and EcpH-ICAM (the membrane marker). EcpH-ICAM consists of the pH-sensitive GFP variant, ecliptic *pH*luorin [47], fused to the transmembrane domain of human ICAM-1 [48]. The incorporation of EcpH-ICAM into the viral membrane enables the visualization of virus entry into mildly acidic compartments based on the loss of the EcpH signal [48,49]. In these experiments, we expressed Rab5 tagged with an orange fluorescent protein mKO [50].

**Figure 4:**
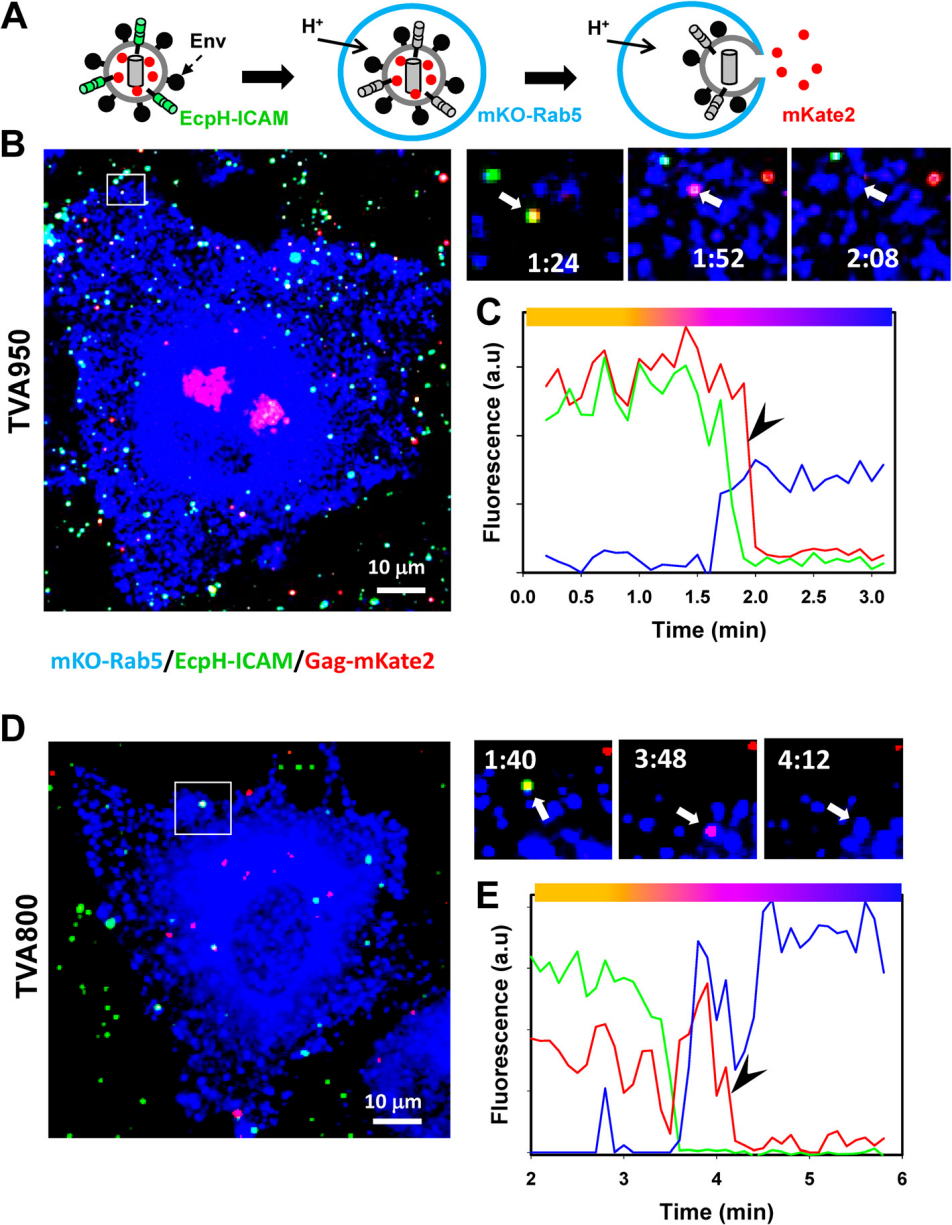
**Single ASLV-A entry into acidic endosomes and virus-endosome fusion. (A)** A diagram illustrating the visualization of the endosomal pH drop and subsequent ASLV-A fusion. **(B-E)** ASLV-A (yellow) fusion with TVA950 **(B, C)** and TVA800 **(D, E)** cells transiently expressing mKO-Rab5 (blue). Pseudoviruses were labeled with EcpH-ICAM (green) and Gag-mKate2 (red). Virus internalization and fusion were initiated by shifting to 37°C at t = 0. **(B, D)** The right top image panels show consecutive snapshots of the enlarged boxed areas showing the virus prior to internalization (left), immediately after entry into acidic Rab5-positive endosomes (middle) and after fusion with early endosomes (right). The graphs in panel C and E show the fluorescence intensities of mKO-Rab5 and the viral EcpH-ICAM (green) and Gag-mKate2 (red) signals as a function of time. The disappearance of the EcpH signal tended to occur concomitantly with ASLV-A/Rab5 colocalization (raise in the mKO signal). Single particle tracking was performed using the mKate2 channel before colocalization with mKO-Rab5, and using the mKO channel afterward. The colored horizontal bars above the graphs in C and E reflect the pseudocolor changes associated with entry of double-labeled particle into Rab5^+^ endosomes, lumen acidification and fusion.

In order to elucidate the relationship between the Rab5 accumulation, endosome acidification and viral fusion, particles co-labeled with EcpH-ICAM (green) and Gag-mKate2 (red) were allowed to enter target cells transfected with mKO-Rab5 (pseudocolored blue, Figure 4A). Entry of double-labeled particles, which appear yellow (Figure 4B,C) into pH-neutral Rab5^+^ endosomes (blue) was not associated with the loss of the EcpH signal, resulting in a transient whitish appearance (not shown). However, virus/Rab5 colocalization without the nearly concomitant loss of the EcpH signal was very brief: the EcpH fluorescence vanished at the time or shortly after Rab5 acquisition, as manifested by the pseudocolor change from yellow or whitish to purple (Figure 4B-E and Additional files 11 and 12: Movies S6 and S7).

**Figure 5:**
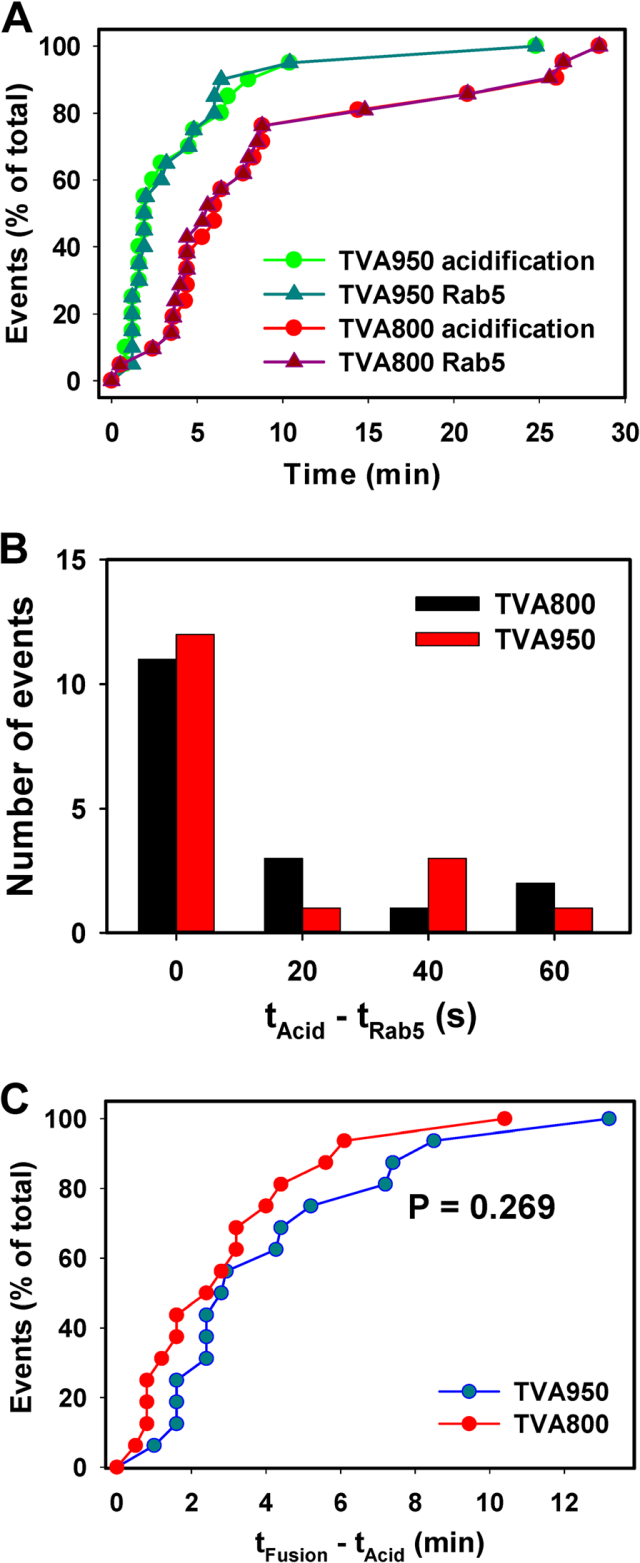
**Kinetics of ASLV-A entry into Rab5 positive endosomes, acidification and fusion. (A)** Distribution of waiting times from raising the temperature (t = 0) to colocalization of double-labeled ASLV-A particles with the mKO-Rab5 signal (triangles) and distribution of waiting times for disappearance of the EcpH signal due acidification of endosomal lumen, (circles) in TVA800 (red/dark red) and in TVA950 (green/dark green) cells. **(B)** Histogram of the time intervals between Rab5 colocalization and EcpH quenching (t_Acid_ – t_Rab5_) in TVA800 and TVA950 cells. **(C)** Kinetics of double-labeled ASLV-A pseudovirus fusion measured as the time interval between the EcpH quenching (acidification) and loss of the mKate2 signal (fusion) in TVA800 and TVA950 cells.

Single particle tracking revealed tight correlation between the appearance of Rab5 and acidification of endosomal lumen in both TVA800 and TVA950 cells. The overall kinetics of the Rab5 accumulation and endosome acidification were superimposable for both TVA isoforms (Figure 5A, P > 0.830). In agreement with our previous results [22], the kinetics of ASLV-A entry into acidic endosomes of TVA950 cells was considerably faster than in TVA800 cells (P = 0.005), reflecting the faster rate of virus uptake through the transmembrane receptor. Analysis of the lag times between Rab5 appearance and acidification for individual virus-carrying endosomes confirmed that these events occurred within a narrow time window (Figure 5B), in good agreement with the previous study [19]. In most cases, we were unable to resolve the lag time between the appearance of Rab5 and disappearance of EcpH signals; the longest delay between the two events was around 60 sec. The distribution of these lag times for TVA800 and TVA950 cells was nearly identical (P = 1.0).

### The kinetics of low pH-dependent ASLV-A fusion with endosomes is independent of TVA isoforms

In spite of the faster kinetics of entry into acidic compartments in TVA950 cells (Figure 5A), the distribution of lags between endosome acidification and viral fusion for each particle was not affected by the TVA isoform (Figure 5C). The acid-induced ASLV-A fusion in TVA800 and TVA950 cells occurred with half-times of 2.2 and 3.2 min, respectively, but the difference between two distributions was not statistically significant. This result is in agreement with our previous measurements of the true kinetics of ASLV-A fusion with TVA-expressing CV-1 cells [36] and with the kinetics of fusion following the virus entry into Rab5^+^ endosomes (Figure 2B). Our findings further support the notion that preferential ASLV-A fusion with early endosomes of TVA950 cells is due to delayed endosome maturation (compare Figures 2B, 3E and 5C). By contrast, the comparable rates of endosome maturation and of low pH-dependent virus fusion in TVA800 cells are most likely responsible for the non-selective entry from early and intermediate compartments in these cells.

## Discussion

Single particle tracking in living cells has revealed that the influenza and Dengue viruses undergo lipid mixing in intermediate and late endosomes, respectively [14,19]. Here, we employed single particle imaging combined with the detection of viral content release into the cytoplasm to define the intracellular sites of ASLV-A entry. These studies showed that ASLV-A releases its content from early or intermediate endosomes, depending on the receptor isoform. Since at least a fraction of full fusion events leads to infection, these findings delineate productive entry pathways of this retrovirus. To our knowledge, this is the first direct demonstration of single virus fusion with specific endosomal compartments using time-resolved live cell imaging.

We found that, unlike the influenza virus, which is sorted to quickly maturing endosomes [19], ASLV-A preferentially enters slowly maturing vesicles in cells expressing TVA950. The virus enrichment in slowly maturing endosomes, combined with the relatively fast rate of low pH-dependent virus fusion, ensures predominant entry from early compartments. By contrast, indiscriminate entry into different pools of vesicles in TVA800 cells results in ASLV-A fusion with both slowly and quickly maturing endosomes. Thus, considering that the Rab5 accumulation and acidification of endosomal lumen are tightly correlated (Figure 5B,C) and that the rate of low pH-dependent ASLV-A fusion is not affected by the receptor isoform (Figure 5C), the sites of entry are likely determined by the kinetic competition between endosome maturation and virus fusion. This notion is supported by the nearly identical kinetics of fusion and maturation in TVA800 cells and considerably slower rate of maturation in TVA950 cells (Figure 3E).

Of note, since a large fraction of pseudoviruses did not co-traffic with either Rab5 or Rab7 (Additional file 3: Figure S3), we cannot rule out the existence of an alternative ASLV-A entry pathway that bypasses Rab5- or Rab7-containing compartments. We consider this possibility unlikely because: (a) a relatively weak fluorescence signal from ectopically expressed Rab5/Rab7 limits our ability to detect small number of these markers on individual endosomes against the high fluorescence background; (b) a stringent co-trafficking criterion applied in this study in order to eliminate spurious colocalization events; (c) the overwhelming majority of non-co-trafficking particles did not exhibit considerable mobility (data not shown) and or lose their content marker, suggesting the lack of entry/fusion activity. These particles thus appear to be non-functional, perhaps devoid of a requisite number of Env glycoproteins.

The above results are in excellent agreement with the predicted sites of ASLV-A entry based on analysis of single particle mobility. We have found that ASLV-A preferentially enters less mobile endosomes in TVA950 cells as compared to TVA800 cells [36]. Considering that endosome mobility tends to correlate with their maturation rate [8,19], we surmised that less mobile virus-harboring compartments corresponded to slowly maturing endosomes. The current study confirmed the correlation between endosome mobility and maturation and demonstrated that ASLV-A and influenza virus have opposite preferences for quickly vs. slowly maturing pools of vesicles. The mechanism of ASLV-A entry into a slowly maturing pool of endosomes in TVA950 cells is currently not understood. Within its cytoplasmic tail, the transmembrane receptor contains two DxF motifs recognized by the AP-2 adaptor, which directs membrane proteins to clathrin-coated pits [51]. It appears, however, that AP-2 does not selectively directs cargo to slowly vs. quickly maturing endosomes, as has been demonstrated for transferrin [36]. It is thus possible that TVA950 non-selectively enters both pools of endosomes, but, because the majority of endosomes are maturing slowly (65%, [36]), most receptor molecules (and therefore most viruses) end up in the latter compartments. In other words, the apparent ASLV-A sorting to slowly maturing endosomes could simply occur through non-selective TVA950 uptake into these compartments. Future studies of TVA800 and TVA950 endocytosis will help delineate the mechanism of selective ASLV-A entry from distinct intracellular compartments.

Endosomal maturation is accompanied by profound changes in their content and membrane composition [9]. These changes are exploited by some viruses to fine-tune their entry sites. For instance, Dengue virus appears to undergo lipid mixing in early endosomes, but requires lyso-bisphosphatidic acid, an unusual lipid concentrated in late endosomes, to undergo full fusion and initiate infection [25]. By contrast, ASLV-A appears to efficiently fuse with both early and intermediate endosomes. The nearly identical fusion kinetics with distinct compartments (Figure 5C), suggests that the lag from endosome acidification to the viral content release is determined by the Env refolding downstream of the receptor priming and low pH-dependent steps [37,38] and not by endosome composition. Interestingly, however, the fraction of pseudoviruses that fused with acidic endosomes was somewhat greater in TVA800 cells compared to TVA950 cells (30% vs. 20%, Table 1). This result indicates that the efficiency of ASLV-A fusion could be affected by the properties of different intracellular compartments. For simplicity, we have not considered here different endosome maturation phenotypes observed in live cell imaging experiments. Consistent with another study [8], at least two maturation patterns were observed for endosomes harboring the virus – gradual accretion of endosomal markers vs. merger with the existing early or intermediate endosomes. It would be interesting to carefully investigate the fate of viruses being routed through different maturation programs.

**Table 1:** Analysis of ASLV-A entry into and fusion with acidic endosomes

**Cell lines**	**Number of EcpH quenching events**	**EcpH quenching events followed by mKate2 release**	**% Fused**
**TVA800**	37	11	29.7
**TVA950**	45	9	20.0

ASLV-A pseudoviruses co-labeled with EcpH-ICAM and Gag-mKate2 were allowed to enter and fuse with TVA800 or TVA950 cells. The number of particles undergoing fusion (mKate2 release) was determined and normalized to the number of particles entering acidic compartments (EcpH quenching).

Another interesting observation is that, under our conditions, ASLV-A particles, even those that did not undergo fusion, did not appear to enter late (Rab5^-^/Rab7^+^) endosomes. Non-fusing pseudoviruses co-trafficked with compartments that did not fully mature in either TVA800 or TVA950 cells for as long as we could track them. Notwithstanding the technical difficulties related to tracking viral particles in highly fluorescent perinuclear area, this result indicates a possible subversion of normal endosomal maturation by ASLV-A. This notion is consistent with the remarkable ability of this virus to retain its fusion competency after a prolonged co-culture with cells in the presence of NH_4_Cl, which blocks virus fusion by raising endosomal pH [34,35,39].

The ASLV-A’s ability to infect cells by fusing with early endosomes shows that the retroviral core can get to the nucleus all the way from the cell periphery. By analogy to HIV-1 [17], retrograde trafficking of the ASLV-A core is likely mediated by cytoskeleton. Replicative advantages conferred by the ASLV-A fusion with early endosomes, as opposed to late endosomes, which bring the virus closer to the nucleus, are currently not clear. Perhaps early escape from endosomes minimizes the chances for virus degradation. Regardless of the reasons for entry from early or intermediate compartments, it is apparent that post-fusion transport of viral cores to the nuclear membrane occurs quite efficiently. Moreover, under certain conditions, retroviral cores can cross the endosomal membrane and enter the cytoplasm. We have obtained evidence that, following the transient NH_4_Cl arrest, the HIV-1 cores pseudotyped ASLV-A Env can be delivered into vacuoles through low pH-dependent fusion [35]. Remarkably, these cores escaped from vacuoles *via* a yet unknown temperature-dependent process, perhaps akin to back-fusion of intralumenal vesicles with the limiting membrane of multivesicular bodies [6]. Back-fusion has been implicated in entry of diverse enveloped viruses [6,52]. Future studies of the retroviral core transport from different cellular locations to the nucleus should shed light on the host factors that are essential for infection.

## Conclusions

Through the visualization of ASLV-A fusion with intracellular compartments tagged by fluorescent markers for early and late endosomes, we pinpointed the sites of viral entry and demonstrated that these sites are regulated by the naturally occurring isoforms of the cognate receptor. Whereas the transmembrane receptor favored ASLV-A fusion with early endosomes, the GPI-anchored isoform directed the viral fusion to intermediate endosomes without delaying the low pH-mediated fusion. The ability to enter from distinct intracellular compartments is conferred by preferential ASLV-A entry into slowly maturing endosomes in cells expressing the transmembrane receptor. Our results also suggest that ASLV-A inhibits maturation of intermediate compartments into late endosomes, perhaps to avoid degradation and maximize the fusion efficiency. These findings provide new insights into retroviral entry pathways and their regulation by cognate receptors.

## Methods

### Cell lines and plasmids

HEK 293 T/17 cells were obtained from ATCC (Manassas, VA) and passaged as described elsewhere [35]. CV-1 cells expressing high levels of the TVA receptor isoforms, CV-1/TVA800 and CV-1/TVA950, have been described previously [35]. The ASLV-A envelope glycoprotein lacking the cytoplasmic domain [33], and MLV Gag-mKate2 and EcpH-ICAM constructs [35,48] have been described previously. Vectors expressing MLV Gag-Pol, MLV LTR lacZ [53] were obtained from Dr. W. Mothes (Yale University), and the pECFP-C1-Rab5 and pEYFP-C1-Rab7 expression vectors [19] were a gift from Dr. X. Zhuang (Harvard University).

### Construction of mKO-Rab5 expression vector

To construct mKO-Rab5 expression vector, mKO gene was amplified by PCR using pmKO-MN1 (Amalgaam MBL, Tokyo, Japan) as template, the forward primer containing *AgeI* restriction site (italicized) 5’-CT*ACCGGT*CGCCACCATGGTGAGTGTGATTAAAC-3’ and the reverse primer containing *HindIII* restriction site (underlined sequence): 5’ – CG*AAGCTT*GGAATGAGCTACTGCATCTTCTAC-3’. The amplified fragment was cloned into pCR4blunt-topo vector using TOPO cloning kit (Invitrogen, Grand Island, NY). After verification of the mKO sequence, the mRFP sequence in the mRFP-Rab5 vector (Addgene, Cambridge, MA) was replaced with the mKO fragment using *AgeI* and *HindIII* restriction sites.

#### *Virus preparation*

Fluorescent pseudoviruses were produced in HEK 293 T/17 cells using PolyFect Transfection reagent (Qiagen, Valencia, CA). Cells grown on a 10 cm dish were transfected with 2 μg MLV-Gag-Pol, 1 μg MLV Gag-mKate2, 3 μg pMLV-LTR-LacZ and 3 μg of the cytoplasmic tail-truncated ASLV-A Env. To introduce a pH-sensor into the viral membrane, 3 μg of EcpH-ICAM-encoding plasmid was added to the DNA transfection mixture. Virus-containing medium was collected 48 h post-transfection, passed through a 0.45 μm filter, aliquoted and stored at -80°C. The infectious titer was determined by a β-galactosidase assay in CV-1 cells expressing TVA800, as described previously [23]

#### *Transient expression of tagged endosomal markers*

2∙10^5^ CV-1 cells stably expressing either TVA950 or TVA800 receptors were seeded on 35 mm Petri dishes (Mattek, Ashland, Massachusetts) in phenol red-free DMEM the day before transfection. On the next day, 80% confluent cells were transfected with 0.5 μg of each CFP-Rab5 and YFP-Rab7 plasmids or mKO-Rab5, using Nanofectin transfection reagent (PAA Laboratories, Dartmouth, MA). The cells were used for imaging 24 h post-transfection.

#### *Imaging virus entry into acidic compartments and fusion*

CV-1/TVA950 or CV-1/TVA800 cells transfected with either CFP-Rab5 and YFP-Rab7 or mKO-Rab5 were placed on ice, washed with cold Hank’s buffer (HBSS), and centrifuged with ~1.5 · 10^4^ IU of single labeled with Gag-mKate2 pseudoviruses or particles co-labeled with EcpH-ICAM and Gag-mKate2, respectively, at 2,100 × g (4°C) for 20 min. Unbound viruses were removed by washing, cells were mounted onto a microscope stage maintained at 37°C. Once a suitable image field was chosen, virus internalization and fusion were initiated by adding 1 ml of warm HBSS and imaged using a Zeiss LSM 780 confocal microscope (Zeiss Microsystems, Jena, Germany) with a 63×/1.4 NA oil immersion objective. Images were acquired every 8–12 sec for ~60 min. The axial position of a specimen during acquisition was stabilized using the Definite Focus module. Cells overexpressing the endosomal markers and/or containing aberrant swollen endosomes were excluded from analysis.EcpH and mKate2 were excited with the 488 and 561 nm laser lines, respectively. CFP-Rab5 and YFP-Rab7 were excited with 458 nm and 514 nm lines, respectively, whereas the 543 nm line was used for mKO-Rab5. Fluorescence emission was detected with the 32-channel GaAsP spectral detector. In experiments with the single labeled virus (Gag-mKate2) entering cells co-transfected with CFP-Rab5 and YFP-Rab7, the first 16 channels recorded the CFP signal and the remaining 16-channels acquired the YFP emission signal, the mKate2 fluorescence was detected using a cooled PMT. When using double-labeled viruses (EcpH-ICAM and Gag-mKate2) and cells expressing mKO-Rab5, the first 16 channels of the GaAsP detector were allocated for the EcpH signal and the remaining 16 channels for the mKO signal, while the mKate2 signal was acquired with the cooled PMT. The emission windows for the fluorescent proteins utilized where selected as follows: CFP (465–500 nm), EcpH (500–540 nm), YFP (520–560 nm), mKO (550–585 nm) and mKate2 (600–650 nm). Spectral unmixing was applied, as necessary, to correct for bleed-through between the CFP and YFP channels and between the EcpH and mKO channels. The above imaging conditions ensured negligible bleed through between CFP and YFP channels (e.g., Figures 1C and 3B).

#### *Image analyses*

Single virus tracking was performed with Imaris (BitPlane, Switzerland) or Volocity (Perkin Elmer, MA) software. Both single- (Gag-mKate2) and double-labeled (EcpH-ICAM and Gag-mKate2) viruses that entered the cell and colocalized with early endosomes (decorated with CFP-Rab5 or mKO-Rab5, depending on the experiment) were tracked using the red channel. The acquisition of Rab5 or Rab7 by virus-carrying endosomes was defined as the point when the endosomal marker signal exceeded the background level by 30%. Virus-endosome colocalization analysis was carried out with ImageJ (http://imagej.nih.gov/ij/). A line histogram that showed normalized intensity profiles of the viral and endosomal markers. The percentage of overlap between the profiles was obtained by calculating the area under the curve for each channel. Viruses showing at least 80% overlap with endosomal markers during >5 consecutive frames and traveled at least 1 μm were considered as co-trafficking with endosomes. After viruses colocalized with endosomal markers for 5 consecutive frames additional tracking was performed, using the CFP-Rab5 signal or the mKO-Rab5 signal as a reference, as indicated. Fusion was detected as disappearance of the red signal (Gag-mKate2 release) whilst the Rab5 signal remained steady. In some cases, quickly traveling endosomes co-localized with the virus were tracked manually using the Volocity software. These analyses yielded the mean fluorescence intensity of viral and endosomal markers, as well as their coordinates, as a function of time. Statistical analysis of the kinetics data was performed using a two-tailed t-test or Rank Sum test, and fraction of viruses fused in cells expressing TVA800 and TVA950 cells was analysed using a χ^2^ test (SigmaPlot, San Jose, CA).

## Competing interests

The authors have no competing interests to declare.

## Authors’ contributions

GBM and SP-P conceived and planned the experiments. SP-P and MM performed the experiments. NK made key fluorescent constructs. SP-P and GBM analyzed the data and co-wrote the manuscript. All authors read and approved the final manuscript.

## Supplementary Material

Additional file 1: Figure S1 Quantification of Rab5 and Rab7 expression in transiently transfected TVA800 and TVA950 cells. Mean CFP-Rab5 **(A)** and YFP-Rab7 **(B)** fluorescence of individual transfected TVA800 and TVA950 cells (both selected and excluded from virus fusion analyses) was determined by selecting regions of interest comprising the whole cell using ImageJ. The background signal was subtracted and spectral bleed-through was eliminated, as described in Methods. The statistical significance of mean fluorescence values for TVA800 and TVA950 cells are shown above the plots. Click here for file

Additional file 2: Figure S2 Determination of virus-endosome colocalization. Line histograms show normalized local maxima for mKate2 (the viral content marker, red) and CFP-Rab5 (blue), as the viral particle merged with an early, CFP-Rab5^+^ endosome. A >80% overlap of areas under line histograms constituted virus-endosome colocalization. Subsequent virus fusion is manifested in the loss of the mKate2 signal (t = 660 s). Click here for file

Additional file 3: Figure S3 Fraction of ASLV-A pseudoviruses cotrafficking with Rab markers and fusing with endosomes. The number of single-labeled (Gag-mKate2) ASLV-A pseudoviruses that co-trafficked with either CFP-Rab5 or YFP-Rab7 and the number of particles that co-trafficked with these markers and released their mKate2 content (i.e., fused) is shown for TVA800 and TVA950 cells. Virus-endosome colocalization was determined, as described in Methods and illustrated in Figure S2. Results of 6 independent experiments for each cell line are shown. Note that the loss of the mKate2 signal from single-labeled viruses which did not colocalize with endosomal markers could not be unambiguously interpreted as fusion. These events could also represent particle detachment from cells or the inability to reliably track particles that shift large distances between consecutive image frames. Click here for file

Additional file 4 **Movie 1.** Single ASLV-A pseudovirus fusion with an early endosome of a TVA950 cell. Fusion of pseudovirus labeled with Gag-mKate2 (red) with TVA950 cells was detected in a CFP-Rab5^+^ endosomal compartment (blue) with no detectable YFP-Rab7 signal (green). The movie is played at 5 frames/sec (40 x speeds). For details, see Figure 1B. Click here for file

Additional file 5 **Movie 2.** Single ASLV-A pseudovirus fusion with a maturing endosome in a TVA800 cell. Pseudovirus containing the Gag-mKate2 (red) marker co-traffics with a Rab5^+^ (blue) endosome and enters a maturing Rab5^+^/Rab7^+^ (blue/green) endosome prior to fusion. Parallel windows showing a combination of red/green/blue (RGB), red/blue (RB) and red/green (RG) channels are shown side-by-side to better illustrate ASLV-A colocalization with markers of early (CFP-Rab5) and late (YFP-Rab7) endosomes. The movie is played at 5 frames/sec (40 x speeds); the frame rate is slower at the end of the movie to better resolve the disappearance of the mKate2 signal. Scale bar for the zoomed region of interest is 4 μm. For details, see Figure 1D. Click here for file

Additional file 6: Figure S4 Examples of Rab5 and Rab7 accumulation by ASLV-A pseudovirus-carrying endosomes in TVA950 cells. ASLV-A pseudoviruses labeled with Gag-mKate2 (red) were pre-bound in the cold to TVA950 cells co-expressing CFP-Rab5 (blue) and YFP-Rab7 (green), and their entry/fusion was initiated by raising the temperature at t = 0. (A, B) Particles that did not undergo fusion accumulate in Rab5^+^/Rab7^+^ compartments. Right panels show the fluorescence intensities of viral (Gag-mKate2) and endosomal (CFP-Rab5 and YFP-Rab7) markers obtained by single particle tracking. The Rab5 and Rab7 accumulation occurred either through an abrupt increase of the Rab5 and Rab7 signals upon virus entry into pre-existing endosomes **(A)** or by gradual accumulation of these markers **(B)**. An intermediate endosome that fuses with virus-bearing endosome at t ~14 min is marked by an arrowhead in panel A. Scale bars are 3 μm **(A)** 5 μm **(B)**. **(C)** Fluorescence intensities showing ASLV-A fusion with an early endosome. Sudden appearance in the Rab5 signal is due to fusion with the existing Rab5^+^ endosome. Click here for file

Additional file 7: Figure S5 Examples of Rab5 and Rab7 accumulation by ASLV-A pseudovirus-carrying endosomes in TVA800 cells. ASLV-A pseudoviruses labeled with Gag-mKate2 (red) were pre-bound in the cold to TVA800 cells co-expressing CFP-Rab5 (blue) and YFP-Rab7 (green), and their entry/fusion was initiated by raising the temperature at t = 0. **(A, B)** Images and intensity profiles for non-fusing particles that co-traffic with Rab5^+^ and Rab^+^/Rab7^+^ endosomes, as determined by single virus tracking. The Rab5 and Rab7 accumulation occurred either in a stepwise fashion (**A**, an early endosome fusing with virus-carrying compartment is shown by an arrowhead) or by gradual accumulation **(B)**. Scale bars are 2 μm (A) and 5 μm **(B)**. **(C)** Fluorescence intensities upon ASLV-A fusion with an early Rab5^+^ endosome. CFP-Rab5 is gradually accumulated in the virus-carrying endosome. Click here for file

Additional file 8 **Movie 3.** ASLV-A fusion with an early endosome in a TVA950 cell and subsequent endosome maturation. Shortly ASLV-A pseudovirus content (Gag-mKate2, red) release occurred from an early, CFP-Rab5^+^ (blue) compartment, endosome maturation ensued, as evidenced by raise of the YFP-Rab7 (green) signal. The movie is played at 5 frames/sec (40x speed). For more information, see Figure 3A. Click here for file

Additional file 9 **Movie 4.** ASLV-A fusion with an early endosome in a TVA800 cell and subsequent endosome maturation. ASLV-A pseudovirus (red) fusion with a TVA800 cell occurs in a highly mobile CFP-Rab5^+^ compartment (blue), followed by a gradual increase of the YFP-Rab7^+^ signal (green). Scale bar 5 μm (first frames). The movie is played at 2 frames/sec (16x speed). For more information, see Figure 3C. Click here for file

Additional file 10 **Movie 5.** Non-fusing ASLV-A pseudovirus traffics through early endosomes and enters intermediate compartments in TVA950 cells**.** Following the internalization of with ASLV-A pseudoviruses containing Gag-mKate2 (red) by TVA950 cells transiently co-expressing CFP-Rab5 (blue) and YFP-Rab7 (green), the virus enters Rab5^+^ and then Rab5^+^/Rab7^+^ endosomes. The movie is played at 5 frames/sec (40x speed). Click here for file

Additional file 11 **Movie 6.** ASLV-A entry into an early endosome and concomitant acidification of endosomal lumen in TVA950 cell. ASLV-A pseudovirus co-labeled with Gag-mKate2 (red) and EcpH-ICAM (green) enters the early mKO-Rab5^+^ endosome (blue) in a TVA950 cell. Due to colocalization of three colors virus-endosome complex appears whitish (arrow). Soon after virus entry, the endosome becomes acidic, as evidenced by the loss of the EcpH-ICAM signal (purple particle), which in turn leads to ASLV-A content release into the cytosol (blue particle). The frame rate is slower around the very brief interval between acidification and fusion. The movie is played at 5 frames/sec (40x speed). For more information, see Figure 4B. Click here for file

Additional file 12 **Movie 7.** ASLV-A entry into an early endosome and concomitant acidification of endosomal lumen in TVA800 cell. ASLV-A pseudovirus co-labeled with Gag-mKate2 (red) and EcpH-ICAM (green) enters the early mKO-Rab5^+^ endosome (blue) in a TVA950 cell. Due to colocalization of three colors virus-endosome complex appears yellowish (arrow). Soon after virus entry, the endosome becomes acidic, as evidenced by the loss of the EcpH-ICAM signal (purple particle), which in turn leads to ASLV-A content release into the cytosol (blue particle). Scale bar for the zoomed region of interest is 5 μm. The movie is played at 5 frames/sec (40x speed). For more information, see Figure 4D. Click here for file
