# Supplementary figures and images for: Pinpointing retrovirus entry sites in cells expressing alternatively spliced receptor isoforms by single virus imaging
Source: Retrovirology. 2014 Jun 16;11:47. doi: 10.1186/1742-4690-11-47 (PMC4065388; doi:10.1186/1742-4690-11-47)

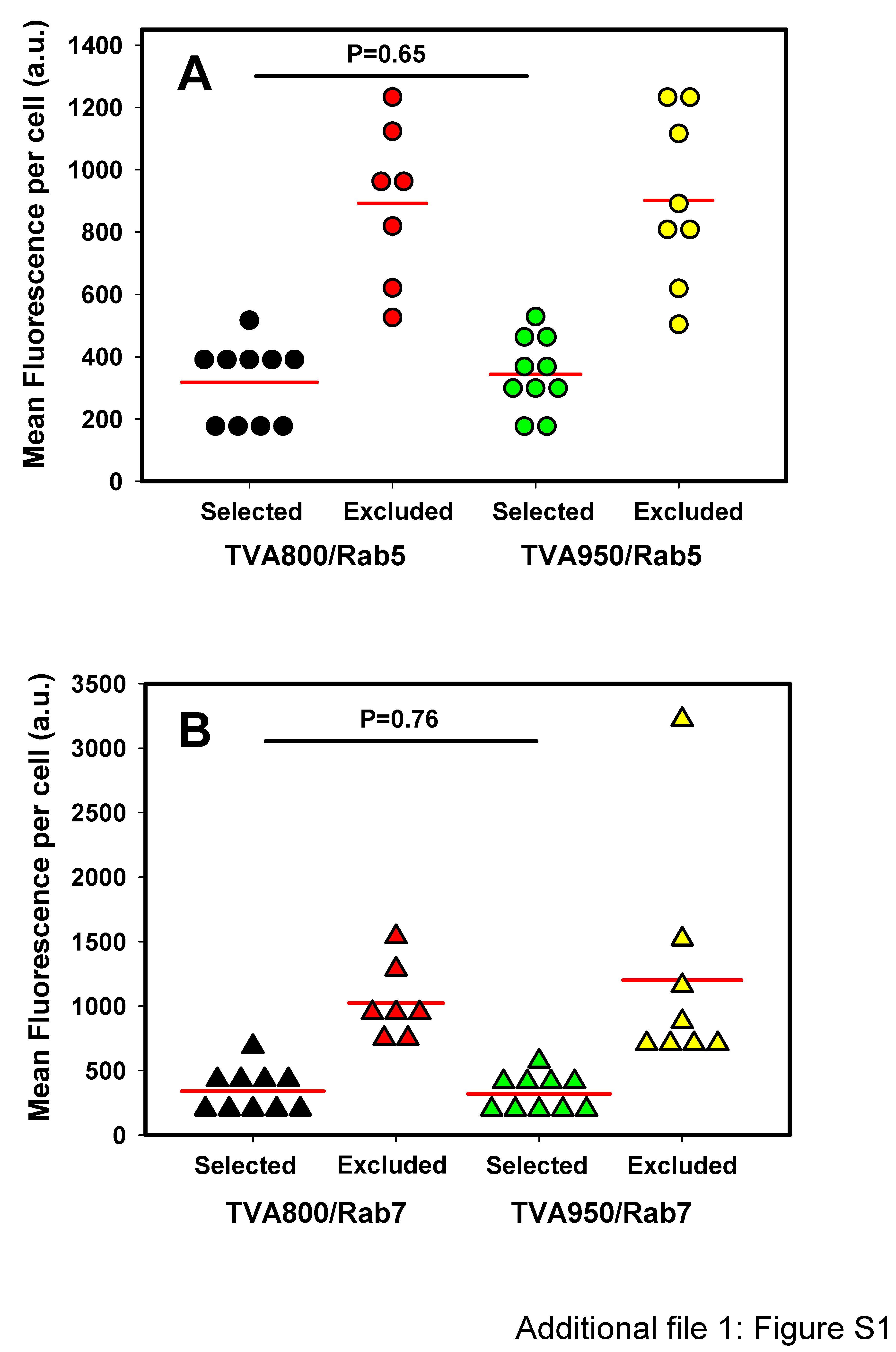

Supplement: Additional file 1: Figure S1 — Quantification of Rab5 and Rab7 expression in transiently transfected TVA800 and TVA950 cells. Mean CFP-Rab5 (A) and YFP-Rab7 (B) fluorescence of individual transfected TVA800 and TVA950 cells (both selected and excluded from virus fusion analyses) was determined by selecting regions of interest comprising the whole cell using ImageJ. The background signal was subtracted and spectral bleed-through was eliminated, as described in Methods. The statistical significance of mean fluorescence values for TVA800 and TVA950 cells are shown above the plots. [file 1742-4690-11-47-S1.tiff]

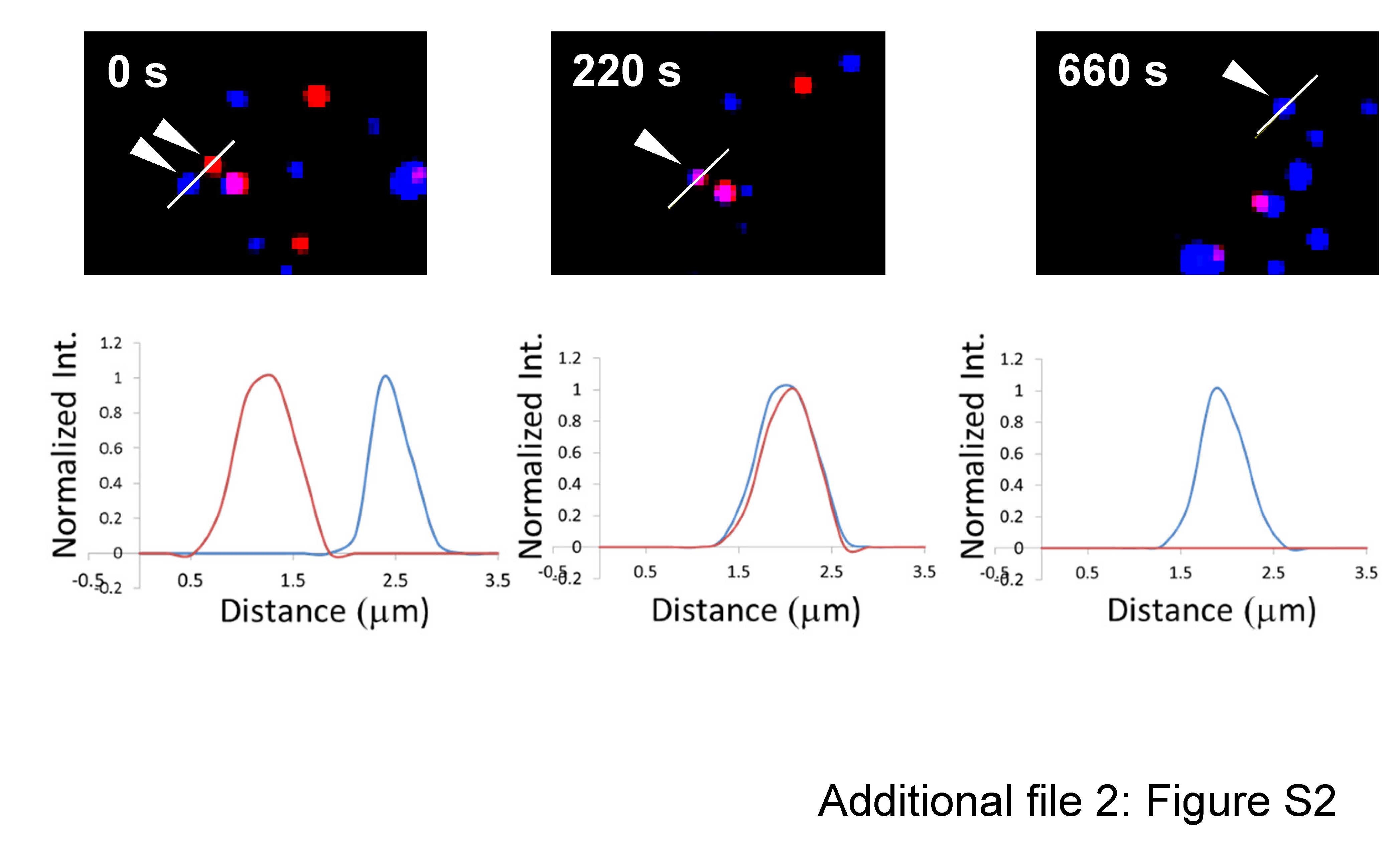

Supplement: Additional file 2: Figure S2 — Determination of virus-endosome colocalization. Line histograms show normalized local maxima for mKate2 (the viral content marker, red) and CFP-Rab5 (blue), as the viral particle merged with an early, CFP-Rab5+ endosome. A >80% overlap of areas under line histograms constituted virus-endosome colocalization. Subsequent virus fusion is manifested in the loss of the mKate2 signal (t = 660 s). [file 1742-4690-11-47-S2.tiff]

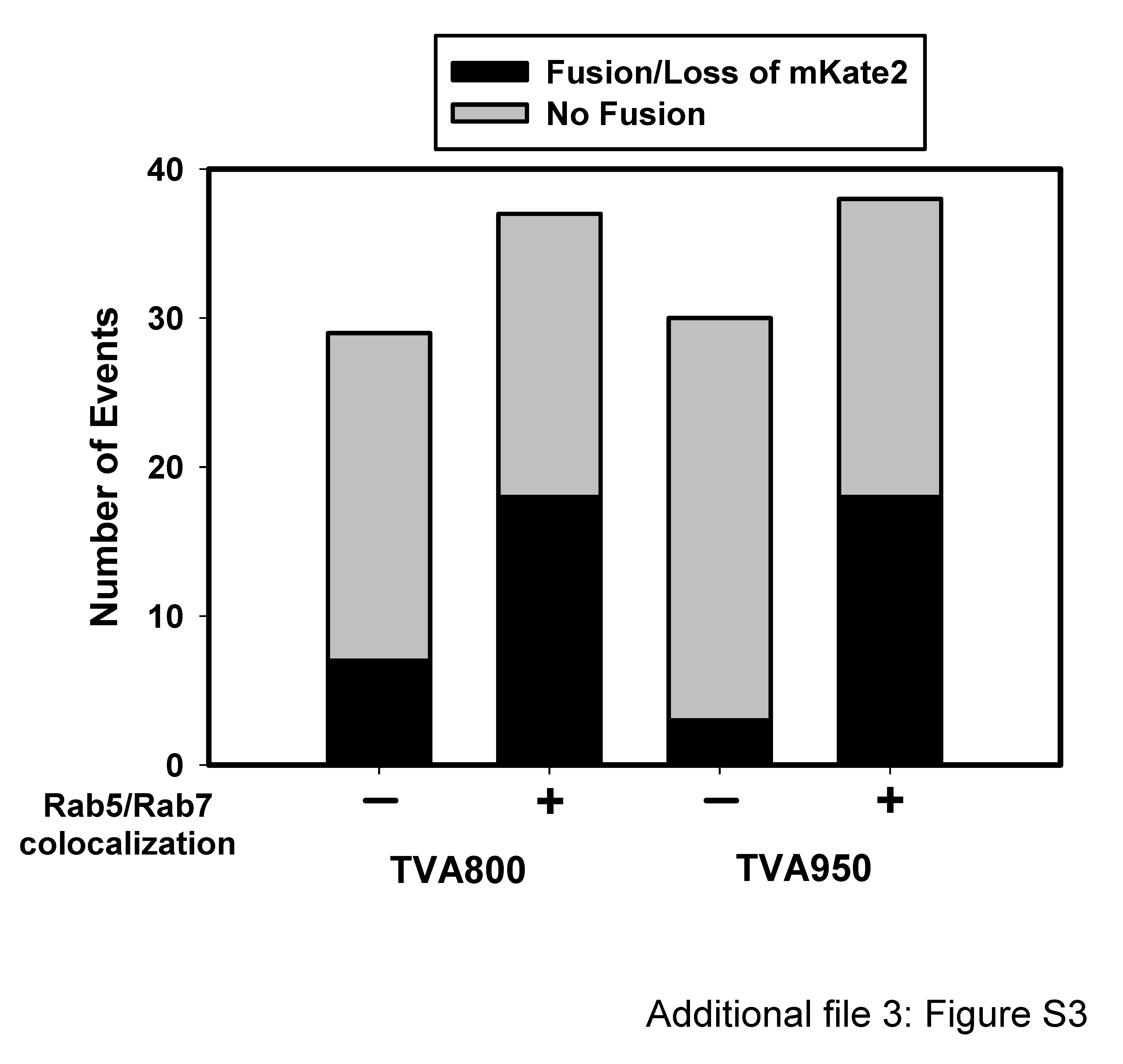

Supplement: Additional file 3: Figure S3 — Fraction of ASLV-A pseudoviruses cotrafficking with Rab markers and fusing with endosomes. The number of single-labeled (Gag-mKate2) ASLV-A pseudoviruses that co-trafficked with either CFP-Rab5 or YFP-Rab7 and the number of particles that co-trafficked with these markers and released their mKate2 content (i.e., fused) is shown for TVA800 and TVA950 cells. Virus-endosome colocalization was determined, as described in Methods and illustrated in Figure S2. Results of 6 independent experiments for each cell line are shown. Note that the loss of the mKate2 signal from single-labeled viruses which did not colocalize with endosomal markers could not be unambiguously interpreted as fusion. These events could also represent particle detachment from cells or the inability to reliably track particles that shift large distances between consecutive image frames. [file 1742-4690-11-47-S3.tiff]

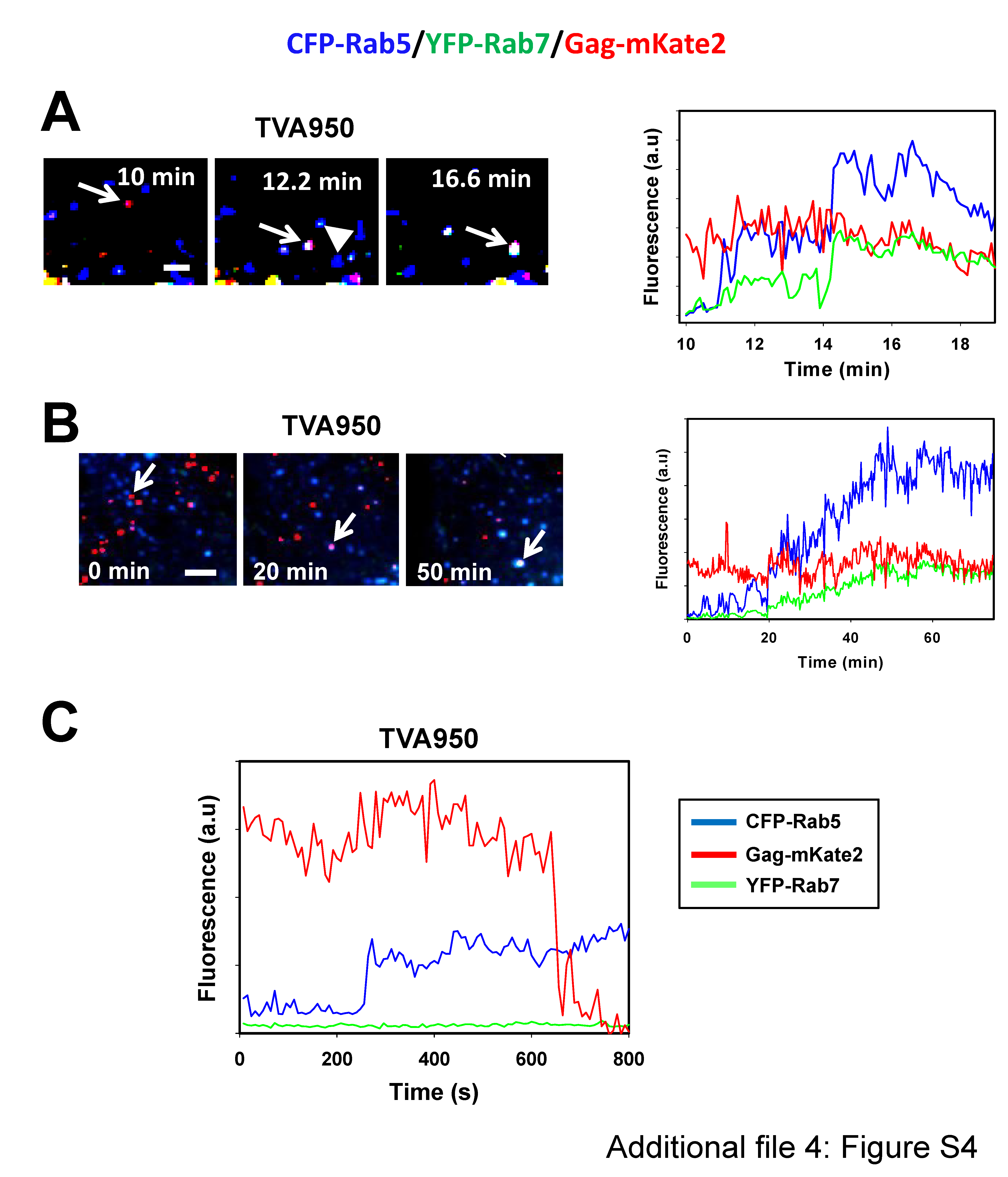

Supplement: Additional file 6: Figure S4 — Examples of Rab5 and Rab7 accumulation by ASLV-A pseudovirus-carrying endosomes in TVA950 cells. ASLV-A pseudoviruses labeled with Gag-mKate2 (red) were pre-bound in the cold to TVA950 cells co-expressing CFP-Rab5 (blue) and YFP-Rab7 (green), and their entry/fusion was initiated by raising the temperature at t = 0. (A, B) Particles that did not undergo fusion accumulate in Rab5+/Rab7+ compartments. Right panels show the fluorescence intensities of viral (Gag-mKate2) and endosomal (CFP-Rab5 and YFP-Rab7) markers obtained by single particle tracking. The Rab5 and Rab7 accumulation occurred either through an abrupt increase of the Rab5 and Rab7 signals upon virus entry into pre-existing endosomes (A) or by gradual accumulation of these markers (B). An intermediate endosome that fuses with virus-bearing endosome at t ~14 min is marked by an arrowhead in panel A. Scale bars are 3 μm (A) 5 μm (B). (C) Fluorescence intensities showing ASLV-A fusion with an early endosome. Sudden appearance in the Rab5 signal is due to fusion with the existing Rab5+ endosome. [file 1742-4690-11-47-S6.tiff]

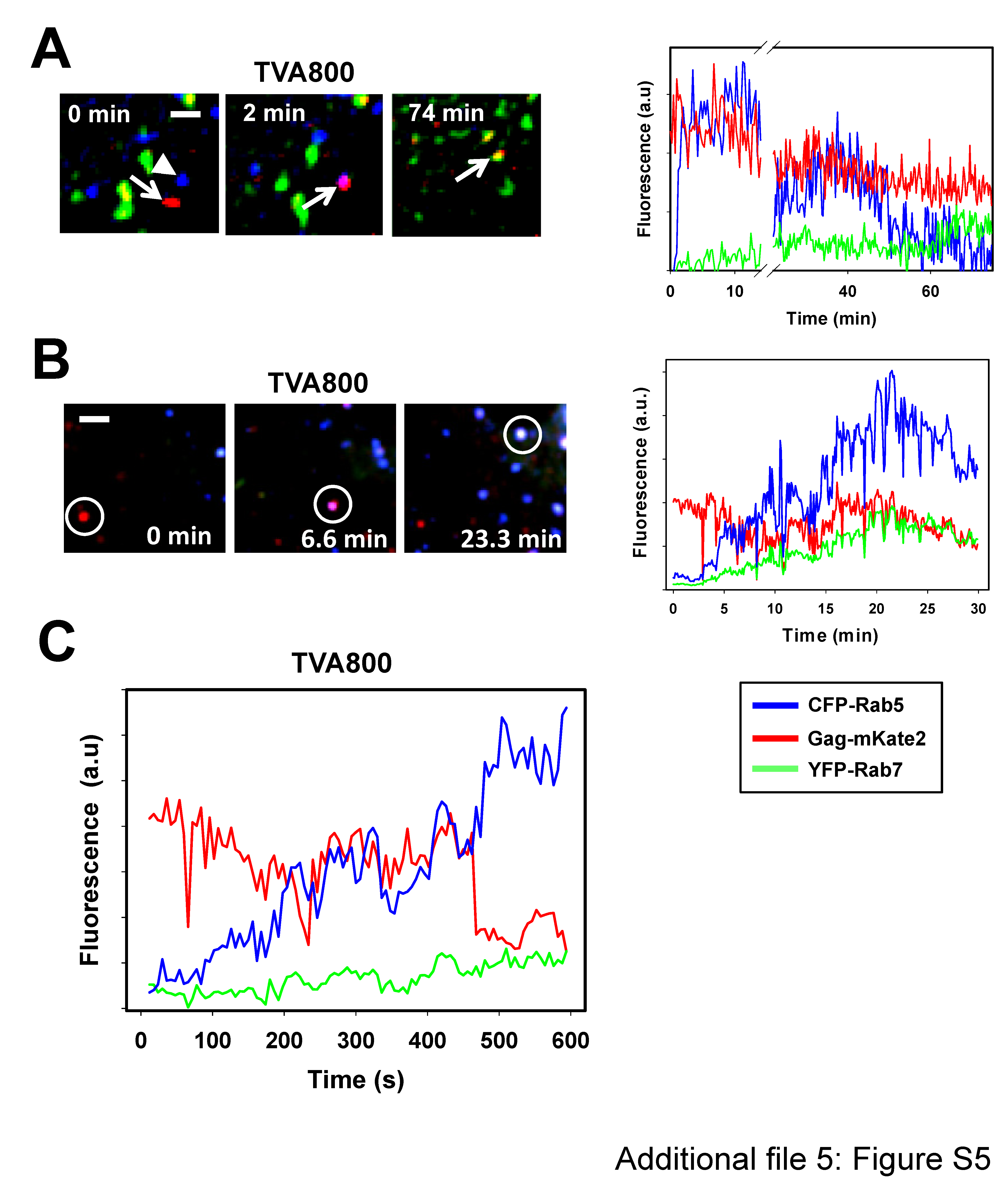

Supplement: Additional file 7: Figure S5 — Examples of Rab5 and Rab7 accumulation by ASLV-A pseudovirus-carrying endosomes in TVA800 cells. ASLV-A pseudoviruses labeled with Gag-mKate2 (red) were pre-bound in the cold to TVA800 cells co-expressing CFP-Rab5 (blue) and YFP-Rab7 (green), and their entry/fusion was initiated by raising the temperature at t = 0. (A, B) Images and intensity profiles for non-fusing particles that co-traffic with Rab5+ and Rab+/Rab7+ endosomes, as determined by single virus tracking. The Rab5 and Rab7 accumulation occurred either in a stepwise fashion (A, an early endosome fusing with virus-carrying compartment is shown by an arrowhead) or by gradual accumulation (B). Scale bars are 2 μm (A) and 5 μm (B). (C) Fluorescence intensities upon ASLV-A fusion with an early Rab5+ endosome. CFP-Rab5 is gradually accumulated in the virus-carrying endosome. [file 1742-4690-11-47-S7.tiff]
